# ADHD prescribing: national findings of children and adolescents attending mental health services in Ireland

**DOI:** 10.1007/s11096-025-01979-z

**Published:** 2025-09-01

**Authors:** Etain Cantwell, Ivana Nelan, Sharifah Zahirah Idid, Suzanne McCarthy, David O. Driscoll

**Affiliations:** 1 School of Public Health, Western Gateway Building, University College Cork, Cork, Ireland; 2 Specialist Neurodevelopmental ADHD Pathway, Cork and Kerry Mental Health Services, Cork, Ireland; 3 School of Pharmacy, University College Cork, Cork, Ireland

**Keywords:** ADHD, Adolescent, Child, Medication, Prescribing

## Abstract

**Introduction:**

Attention deficit hyperactivity disorder (ADHD) is a neurodevelopmental condition, which initially presents in childhood. While prescribing trends for treating ADHD have been previously examined in Ireland’s paediatric population, off-label prescribing of ADHD medication has yet to be studied.

**Aim:**

We aimed to describe ADHD medication prescribing and off-label prescribing of ADHD medication in Ireland.

**Method:**

This cross-sectional study used a sample drawn from the population of children and adolescents who were attending mental health services in Ireland as of 31st December 2021. Participants were included based on predefined inclusion and exclusion criteria; those who were aged 17 years or younger and had been prescribed at least one psychotropic medication (n = 3,193). We described the frequency and population of those prescribed an ADHD stimulant or non-stimulant medication and the target condition or target symptom cluster for prescription. We reported the starting and maintenance doses of each medication.

**Results:**

Fifty-three percentage (n = 1,687) of children and adolescents were prescribed an ADHD medication on 31st December 2021, with more boys being prescribed an ADHD medication compared to girls (n = 1,284 vs n = 395). The most common age category prescribed ADHD medication was 11–13 years of age. The most common indication for prescribing ADHD medication was the target condition ADHD (n = 1,661; 98.5%).Twenty-six patients (1.5%) were prescribed ADHD medication for target symptoms, most commonly depressive (n = 8) and behavioural disturbance symptoms (n = 8) (i.e., off-label prescribing of ADHD medication).

**Conclusion:**

More than half of young people attending specialist mental-health services in Ireland were receiving an ADHD-specific medicine, and almost all prescriptions were tied to a confirmed ADHD diagnosis. Off-label use for other symptom clusters was rare (< 2%), indicating strong adherence to licensed indications but also highlighting the importance of continued surveillance to detect emerging off-label trends and to ensure prescribing remains evidence-based and patient-centred.

**Supplementary Information:**

The online version contains supplementary material available at 10.1007/s11096-025-01979-z.

## Impact statements

- By demonstrating that 98% of ADHD medication prescriptions in Irish CAMHS are on-label, this study provides a measurable standard against which individual services and future audits can evaluate prescribing.
- This study aids as a guide resource for policy planning as it highlights that one in every two child and adolescent mental health service (CAMHS) service users receive ADHD related medication, which further highlights the need for dedicated ADHD services.
- For the families of children and adolescents attending specialist services, these results offer reassurance regarding the quality and safety of ADHD medication management.

## Introduction

Attention-deficit hyperactivity disorder (ADHD) is a heritable, complex neurodevelopmental disorder that manifests during childhood [1]. The diagnosis of ADHD is based on specific criteria outlined in either the International Classification of Diseases, Eleventh Revision (ICD-11) or the Diagnostic and Statistical Manual of Mental Disorders, Fifth Edition (DSM-5) [2, 3]. Both classification systems require persistent symptoms of inattention, hyperactivity or impulsivity presenting at developmentally inappropriate levels and causing significant impairment across multiple settings. Differences between these symptoms include the number of diagnostic criteria, diagnostic thresholds and partioning of hyperactivity and impulsivity into subdimensions [4]. Management of ADHD involves a multimodal treatment strategy, using both non-pharmacological and pharmacological interventions, which alone or in combination, have demonstrated efficacy and tolerability. The National Institute for Health and Care Excellence (NICE) guidelines recommend pharmacological treatment for children aged 5 years and over when ADHD symptoms cause significant and persistent impairment in daily functioning with methylphenidate (either short or long acting) as first line [5].

Previous research has examined prescribing trends of ADHD medications in Ireland. A national cohort study of a paediatric population found a significant increase in the rate of psychostimulant prescribing between 2002 to 2011 with methylphenidate being the most frequently prescribed psychostimulant [6]. A more recent study using the Irish pharmacy claims database reported a 29% increase in the prevalence of dispensed ADHD medications among children and adolescents 5–15 years of age between 2017 to 2021, measured per 1000 eligible individuals in the population. The prevalence of dispensed ADHD medications was higher among males compared to females [7]. Despite this notable upward trend, ADHD medication prescribing and consumption rates in Ireland remain low compared to international figures [7]. Off-label prescribing of ADHD medications refers to the use of medication outside of its approved license and has yet to be examined comprehensively in the Irish paediatric population. An observational cohort study and retrospective analysis of ADHD medication prescriptions in the United States illustrated off-label ADHD medication prescriptions among children, who are younger than the age indicated on medication licenses [8]. Boland and colleagues also described the off-label use of psychostimulants in Ireland in relation to age criteria, although they did not provide the specific indications for which these medications were prescribed [6].

Mental health care in Ireland for children and adolescents up to and including 17 years of age is provided by the child and adolescent mental health service (CAMHS). This is a publicly funded service which provides sectorised care within designated geographical areas. Each CAMHS team comprises a multidisciplinary team, led by a clinical lead consultant child and adolescent psychiatrist. The majority of children and adolescents attending CAMHS receive non-pharmacological interventions, while some may also be prescribed psychotropic medication as part of their treatment [9]. Psychotropic medication can be prescribed by four types of clinicians in Ireland: general practitioners (GP), paediatricians, nurse prescribers, and child and adolescent psychiatrists. The “Maskey Report” published in January 2022 highlighted several issues in the area of governance, clinical care and administrative practices in a specific CAMHS team in Ireland [10]. This report identified the overuse of psychotropic medication with specific reference to off-label use. Due to prescribing safety concerns, the Irish government commissioned a national audit of psychotropic prescribing practices in CAMHS, which aimed to describe the medications prescribed within the service, evaluate the standard of prescribing care and identify the indications for medications prescribed [11].

### Aim

Thus, utilizing data collected from a cross-sectional survey of children and adolescents attending CAMHS in Ireland, the objectives of this study were: (i) to describe ADHD medication prescribing and (ii) to examine off-label prescribing of ADHD medication.

## Method

### Data

In 2021, a national audit was undertaken to evaluate psychotropic prescribing practices across all public child and adolescent mental health services (CAMHS) in Ireland. This audit aimed to capture the types of medications being prescribed, the clinical rationale for their use, and how prescribing aligned with expected standards of care. In total, 74 CAMHS services were included for analysis. A total of 21 081 were attending CAMHS services in July to December 2021 and 3 528 were eligible based on inclusion criteria (i.e., attending the service, 17 years of age or under, active attendance (i.e., attending appointments and/or receiving intervention) between July 2021 and December 2021 and prescribed any psychotropic medication during the above agreed timeline). The analysed sample was limited to those prescribed psychotropic medication on 31st December 2021 (n = 3,193).

According to guidance from the Health Service Executive (HSE), services with more than 500 eligible individuals were required to provide a 10% sample, while services with fewer than 500 participants submitted up to 50 cases for analysis [12]. This approach represents 1 in 6 children attending the CAMHS services in Ireland during the study time period. A standardized data collection tool, developed and tested in advance, was used by participating teams. Completed forms were either uploaded digitally via Smart Survey or submitted manually, depending on the service’s preference and resources.

### Baseline characteristics

The following baseline characteristics were obtained: gender (male, female, other), duration attending the CAMHS services (years, months), consultant in service (yes, no), age (years, months), referral type (urgent, routine), and mental disorder diagnosis provided as (yes, no) (anxiety disorder, depressive disorder, attention deficit hyperactivity disorder (ADHD) or attention deficit disorder (ADD), psychotic disorder, bipolar affective disorder (BPAD), Tourette’s or Tic disorder, eating disorder, obsessive compulsive disorder (OCD). The above listed conditions were outlined in the audit tool, provided in supplementary data, and additional information were obtained from a free text “other diagnosis” section about participants which include autism spectrum disorder, oppositional/conduct symptoms, and aggressive symptoms. Ethnicity and socioeconomic status were not recorded.

### Prescribing standards

The audit assessed six aspects of prescribing quality: (1) Documentation confirming informed consent from a parent or guardian; (2) Confirmation that prescribing decisions were either made or approved by a consultant psychiatrist; (3) Completion of baseline physical health monitoring before initiating medication; (4) Ongoing physical health monitoring while on medication; (5) Communication with the child’s general practitioner regarding treatment; (6) Scheduling of follow-up appointments post-prescription. Documenting side-effects was not recorded as a standard in the audit.

### ADHD medication

The following information on ADHD medication was obtained: medication name, target condition, target symptoms (a target symptom was described in the absence of a target condition by the treating team), starting dose, maintenance dose, starting date of medication, and discontinuation date of medication.

### Off-label prescribing

Off-label prescribing in this study was defined as ADHD medication prescribed for target symptoms, and not ADHD diagnosis, or if ADHD medication was prescribed outside of licensed age range.

### Statistical analysis

Descriptive statistics are presented as numbers and percentages for categorical variables and mean ± standard deviations for continuous variables. Medical group difference was assessed using Pearson’s chi-squared test. The level of statistical significance was set at p < 0.05. We report the number of those prescribed a stimulant or non-stimulant ADHD medication for ADHD condition and the number and percentages of those prescribed a stimulant or non-stimulant medication for target symptom cluster. We report starting and maintenance dose for each medication (sample size, mean, standard deviation, median and minimum to maximum). Statistical analysis was completed using Stata software (v.18).

### Ethics approval

Ethics approval was granted by the Clinical Research Ethics Committee of the Cork Teaching Hospital by authors to complete secondary analysis of the audit data (CREC Review Reference Number and approval/renewal dates: ECM 4(p) 12/09/2023 & ECM 3(f) 05/03/2024). The data controller (HSE) provided permission and released data to the authors.

## Results

### Summary of demographics

The analysed study sample comprised 3,193 children and adolescents attending child and adolescent mental health services in Ireland on 31st December, 2021. Over 46 percentage (n = 1,492) of children and adolescents were prescribed a stimulant medication, over six percentage (n = 195) were prescribed a non-stimulant medication and 47 percentage (n = 1,506) were prescribed other psychotropic medications (Table 1). More boys than girls were prescribed a stimulant (n = 1,144, 76.7% vs n = 342, 22.9%,) and non-stimulant (n = 140, 71.8% vs n = 53, 27.2%) medication (p < 0.001). Conversely, fewer boys than girls were prescribed other psychotropic medications (n = 501, 33.3% vs n = 979, 65%) (p < 0.001). A summary of demographics of children and adolescents presented by gender is provided in supplementary material (Table S1, Table S2).

**Table 1:** Summary of demographics of participants prescribed other psychotropic, stimulant, or non-stimulant medication on 31st December 2021 in Ireland (n = 3,193)

	Other Psychotropic		Stimulant		Non-stimulant		p-value*
	(n = 1,506)		(n = 1,492)		(n = 195)		
	n	(%)	n	(%)	n	(%)	
*Gender of child participant*							< 0.001
Female	979	(65)	342	(22.9)	53	(27.2)	
Male	501	(33.3)	1144	(76.7)	140	(71.8)	
Other	26	(1.7)	6	(0.4)	2	(1)	
*Age categorised (years)*							< 0.001
5–7	8	(0.5)	30	(2)	2	(1)	
8–10	42	(2.8)	268	(18)	43	(22.1)	
11–13	201	(13.3)	489	(32.8)	64	(32.8)	
14–15	447	(29.7)	377	(25.3)	50	(25.6)	
16–17	808	(53.7)	328	(22)	36	(18.5)	
*Categorised duration in service (years)*							< 0.001
0–2	1227	(81.5)	658	(44.1)	63	(32.3)	
3–5	211	(14)	454	(30.4)	71	(36.4)	
6–8	40	(2.7)	248	(16.6)	48	(24.6)	
9–12	19	(1.3)	115	(7.7)	11	(5.6)	
$\ge$ 13	7	(0.5)	14	(0.9)	2	(1)	
Uncodable	2	(0.1)	3	(0.2)	0	(0)	
*Consultant present on the team*							0.70
Yes	1412	(93.8)	1391	(93.2)	183	(93.8)	
*Referral type*							< 0.001
Routine	743	(49.3)	1324	(88.7)	175	(89.7)	
Urgent	754	(50.1)	159	(10.7)	18	(9.2)	
*Moderate—severe diagnosis*							
Anxiety—Yes	843	(56)	96	(6.4)	24	(12.3)	
ADD/ADHD—Yes	166	(11)	1478	(99.1)	190	(97.4)	
Depressive Disorder—Yes	568	(37.7)	31	(2.1)	6	(3.1)	
Eating Disorder—Yes	155	(10.3)	5	(0.3)	1	(0.5)	
OCD—Yes	143	(9.5)	11	(0.7)	3	(1.5)	
psychosis—Yes	43	(2.9)	1	(0.1)	0	(0)	
BPAD—Yes	15	(1)	0	(0)	0	(0)	
Tourette's/ Tics—Yes	23	(1.5)	9	(0.6)	7	(3.6)	
*Medication prescription*							
Methylphenidate	–	–	1295^1^	(86.8)	–	–	
Lisdexamfetamine	–	–	151^2^	(10.1)	–	–	
Atomoxetine	–	–	–	–	117	(60)	
Guanfacine	–	–	–	–	63	(32.3)	
Clonidine	–	–	9	(4.6)	–	–	
*Baseline physical parameters prior to medication*							< 0.001
No	149	(9.9)	101	(6.8)	7	(3.6)	
Yes	839	(55.7)	1371	(91.9)	186	(95.4)	
Not Applicable	518	(34.4)	20	(1.3)	2	(1)	
*Monitor physical parameters during medication*							< 0.001
No	143	(9.5)	66	(4.4)	5	(2.6)	
Yes	791	(52.5)	1421	(95.2)	189	(96.9)	
Not Applicable	572	(38)	5	(0.3)	1	(0.5)	
*Correspondence sent to family doctor (e.g., GP)*							< 0.001
No	187	(12.4)	104	(7.0)	13	(6.7)	
Yes	1319	(87.6)	1388	(93.0)	182	(93.3)	
*Follow up arranged*							0.042
Yes	1481	(98.3)	1458	(97.7)	190	(97.4)	

ADD attention deficit disorder, ADHD attention deficit hyperactivity disorder, OCD obsessive compulsive disorder, BPAD bipolar affective disorder, GP general practitioner (i.e., family doctor)

*Dexamfetamine (n = 1) ^1^Uncodable methylphenidate n = 30 (15.4%). ^2^Uncodable Lisdexamfetamine n = 9 (4.6%)

*Pearson’s chi–squared test, statistical significance p < 0.05

The most common age category prescribed stimulant or non-stimulant medications was 11–13 years of age (Table 1). Almost 90 percentage of children or adolescents prescribed an ADHD medication were routinely referred to the service. Over 99 percentage (n = 1,478) of those who were prescribed a stimulant and 97.4 percentage (n = 190) of those who were prescribed a non-stimulant had a diagnosis of moderate to severe ADD or ADHD.

### Prescribing standards

Children and adolescents prescribed stimulant and non-stimulant medication had high prescribing standards for consultant involved in prescribing (n = 1,391, 93.2% vs n = 183, 93.8%), correspondence to family doctor (n = 1388, 93% vs n = 182, 93.3%), and follow-up arranged (n = 1458, 97.7% vs n = 190, 97.4%). Baseline and ongoing monitoring of physical parameters were more frequently conducted among those prescribed stimulants (baseline n = 1,371, 91.9%, ongoing n = 1,421, 95.2%) and non-stimulants (baseline n = 186, 95.4%, ongoing n = 189, 96.9%) compared to individuals prescribed other psychotropic medication (baseline n = 839, 55.7%, ongoing n = 791, 52.5%) in the service.

### Target conditions and target symptoms of those prescribed any ADHD medication (n = 1687)

A total of 1,687 children and adolescents were prescribed an ADHD-related medication on 31st December 2021 (Table 2). Of those, 87.3 percentage (n = 1,473) were prescribed a stimulant and 11.1 percentage (n = 188) were prescribed a non-stimulant medication for the condition ADHD. Nineteen (1.1%) children and adolescents were prescribed a stimulant and seven (0.4%) were prescribed a non-stimulant for target symptoms. Among those prescribed ADHD medication for target symptoms, the most common symptom clusters were depressive symptoms (n = 8) and behavioural disturbance symptoms (n = 8).

**Table 2:** Target condition and target symptom cluster for children and adolescents prescribed a stimulant or non-stimulant medication on 31st December 2021 in Ireland (n = 1,687)

	Stimulant		Non-stimulant	
	(n = 1,492)		(n = 195)	
Target indication		n (%)		
By condition – ADHD	1,473 (98.7)		188 (96.4)	
By target symptom cluster	19		7	

Breakdown of Target Symptom Cluster	n	(%)^1^	n	(%)^1^
ADHD symptoms yet not diagnosis	1	(0.1)	0	(0)
Anxiety symptoms	0	(0)	1	(0.5)
Aggression/irritability/challenging symptoms	1	(0.1)	0	(0)
Depressive symptoms	8	(0.5)	0	(0)
Behavioural Disturbance symptoms	7	(0.5)	1	(0.5)
Insomnia symptoms	0	(0)	1	(0.5)
Emotional regulation	1	(0.1)	0	(0)
Tic symptoms	0	(0)	3	(1.5)
Documented N/A	1	(0.1)	1	(0.5)

ADHD attention deficit hyperactivity disorder; N/A not applicable

^1^Percentage of target symptom cluster (. e.g., anxiety symptoms) by total target symptom cluster (n = 26)

### Dose of ADHD medication (n = 1690)

The most common ADHD medication prescribed was methylphenidate, with Concerta (n = 348, 20.5%) being the most frequently recorded brand (Table 3). Median starting doses for stimulant products clustered between 10 and 20 mg and were titrated two- to three-fold for maintenance (e.g., Lisdexamfetamine from 20 to 40 mg). Non-stimulants followed a comparable pattern: atomoxetine increased from 10 to 40 mg and guanfacine from 1 to 2 mg, while clonidine doses were more variable. (Table 3).

**Table 3:** Description of starting (n = 1,690) and maintenance (n = 1,684) doses for available stimulant and non-stimulant medications (milligrams, mg) prescribed on 31st December 2021 in Ireland (n = 1,687)

Medication	(n)	Mean	SD	Median (IQR)	Min	Max
*Stimulant*						
Equasym XL						
Starting dose^1^	(262)^1^	14.5	8.8	10 (10)	2.5	60
Maintenance dose^2^	(264)^2^	30.7	12	30 (20)	10	60
Concerta XL						
Starting dose^1^	(348)^1^	23.4	12.1	18 (9)	18	126
Maintenance dose^2^	(351)^2^	40.9	15.4	36 (27)	18	126
Medikinet IR/MR						
Starting dose^1^	(264)	10.8	12.7	10 (5)	2.5	80
Maintenance dose^2^	(259)	26.7	63.9	20 (20)	5	100
Ritalin IR/XL						
Starting dose^1^	(158)	12.4	8.3	10 (15)	2.5	45
Maintenance dose^2^	(156)	26.7	15.2	20 (22.5)	5	80
Methylphenidate IR/XL/LA						
Starting dose^1^	(313)	10.8	9.2	10 (5)	2.5	90
Maintenance dose^2^	(311)	25.5	15.5	20 (15)	5	126
Lisdexamfetamine						
Starting dose^1^	(159)	25.5	8.6	20 (10)	10	80
Maintenance dose^2^	(157)	40.3	16.9	40 (20)	20	120
*Non-stimulant*						
Atomoxetine						
Starting dose^1^	(116)	16.7	10	10 (10)	4	60
Maintenance dose^2^	(116)	38.6	17.2	40 (25)	10	90
Guanfacine						
Starting dose^1^	(61)	1.1	0.6	1 (0.5)	0.5	4
Maintenance dose^2^	(61)	2.5	1.2	2 (1)	1	7
Clonidine						
Starting dose^1^	(9)	41.7	25	25 (25)	25	100
Maintenance dose^2^	(9)	95.6	75.5	100 (50)	0.125	275

SD standard deviations, IQR interquartile range. Available data (n =), missing or uncodable (n =), ^1^ Starting dose documented, ^2^Maintenance dose documented. Dexamfetamine not reported (n = 1)

^1^Equasym XL starting (n = 9) missing or uncodable. ^2^Equasym XL maintenance (n = 7) missing or uncodable

^1^Concerta XL starting (n = 6) missing or uncodable. ^2^Concerta XL maintenance (n = 5) missing or uncodable

^1^Medikinet IR/MR starting (n = 8) missing or uncodable. ^2^Medikinet IR/MR maintenance (n = 10) missing or uncodable

^1^Ritalin IR/XL starting (n = 3) missing or uncodable. ^2^Ritalin IR/XL maintenance (n = 3) missing or uncodable

^1^Methylphenidate IR/XL/LA starting (n = 15) missing or uncodable. ^2^Methylphenidate IR/XL/LA maintenance (n = 15) missing or uncodable

^2^Lisdexamfetamine maintenance (n = 3) missing or uncodable

^1^Atomoxetine starting (n = 1) missing or uncodable. ^2^Atomoxetine maintenance (n = 1) missing or uncodable

^1^Guanfacine starting (n = 2) missing or uncodable. ^2^ Guanfacine maintenance (n = 2) missing or uncodable

### ADHD medication and polypharmacy

**Fig. 1:**
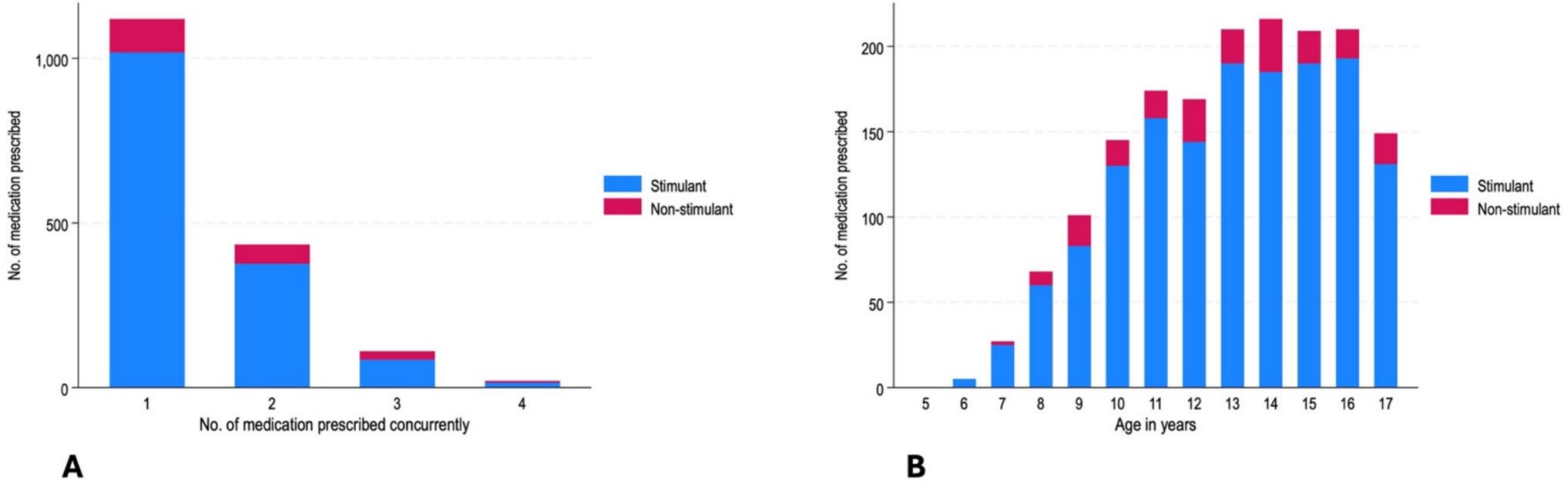
Frequency of stimulant and non-stimulant medication prescriptions by polypharmacy (**a**) and by age (**B**) prescribed on 31st December 2021 (n = 1,687)

**Fig. 2:**
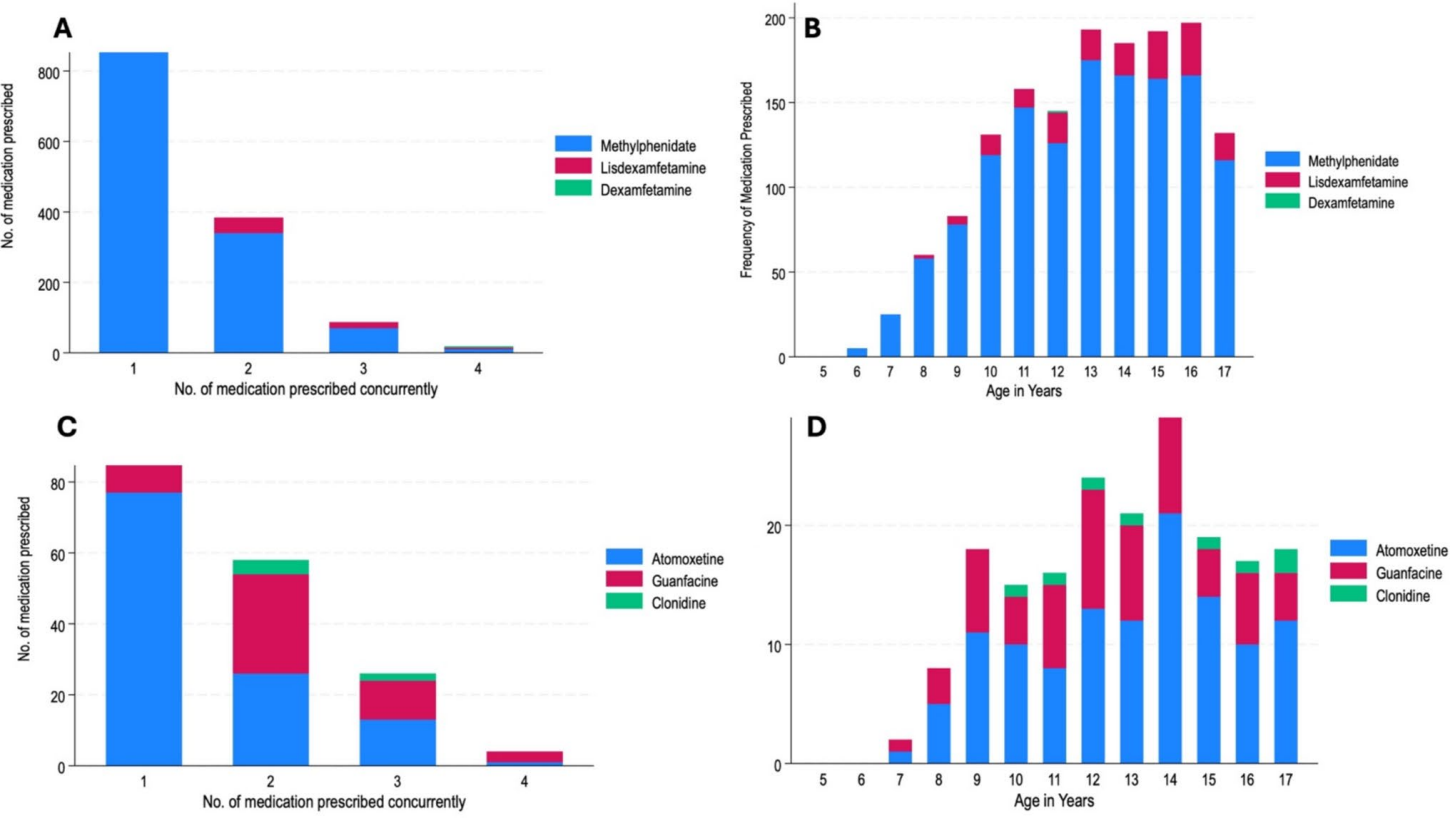
**a** Number of medications prescribed with a stimulant ADHD medication (**b**), age of patients prescribed stimulant ADHD medication (**c**), Number of medications prescribed alongside a non-stimulant ADHD medication (**d**), and age of patients prescribed non-stimulant ADHD medication on 31st December 2021 (n = 1,687)

Most stimulant medication was prescribed without additional co-prescriptions of a further psychotropic (n = 1,018, 68.1%). A small number of children and adolescents were prescribed three (n = 85, 5.7%) or four (n = 15, 1.0%) medications with a stimulant at the same time. Similarly, non-stimulant medication was prescribed without additional co-prescriptions of a further psychotropic (n = 101, 53.4%). Again, a small number of children and adolescents were prescribed three (n = 25, 13.2%) or four (n = 5, 2.7%) medications with a non-stimulant medication at the same time (Figs 1, 2).

## Discussion

This is the first study using a large national sample to describe ADHD medication prescribing practices for children and adolescents attending mental health services in Ireland on 31st December 2021. Our study revealed that forty-six percentage (n = 1,492) of the study sample were prescribed a stimulant, and six percentage (n = 195) were prescribed a non-stimulant medication. We illustrate that stimulant and non-stimulant medication prescriptions were highest in the 11–13 years of age group and the main indication for prescribing an ADHD medication was to treat an ADD/ADHD diagnosis. We report the starting doses and maintenance doses prescribed by clinicians, noting their indications whether to target ADD/ADHD diagnosis or to target specific symptom clusters. Finally, we report a high adherence to standards of good prescribing reported.

The data present minimal amount of off-label prescribing of ADHD medication to children and adolescents attending mental health services in Ireland. It is acknowledged that off-label prescribing is often necessary in paediatric settings due to the limited availability of paediatric dosing information [13]. Off-label prescribing in paediatric settings may occur due to age restrictions, or restrictions in indications, dosage or routes of administration [13]. NICE guidelines do not recommend the use of drug treatment with ADHD medications for those aged 5 years and under without obtaining a second opinion from an expert ADHD service that specializes in managing ADHD in young children, preferably a tertiary-level care service [5]. However, a study by Panther and colleagues found that over 91 percentage of ADHD medication prescribed to children aged 3–5 years were off-label, as this age cohort were younger than the age indicated on medication licenses [8]. Boland and colleagues examined psychostimulant prescribing trends in a paediatric population in Ireland from 2002 to 2011, using data from the General Medical Services (GMS) scheme pharmacy claims database [6]. A low prescribing rate of methylphenidate was observed in the under four years of age cohort. ADHD medication prescriptions were highest among those 11–13 years of age in our study cohort. This is consistent with previous findings in the Irish paediatric setting, which found prescribing rates highest among those aged 12–15 years (Boland et al., 2015; Mac Avin et al., 2020). In the United Kingdom, ADHD medication prescribing was noted to be most prevalent among those aged 6–12 years [15]. These trends align with international literature. In Germany, ADHD medication initiation was most frequently reported among children and adolescents below 16 years of age, with a peak at 10 years of age [16]. Our cohort presented low numbers in the age category between 5–7 years of age being prescribed either a stimulant (n = 30, 2%) or non-stimulant medication (n = 2, 1%), and no data were available on medications prescribed under 5 years of age. More boys than girls were prescribed stimulant and non-stimulant medication. This finding is consistent with previous research in the Irish paediatric setting which reported a higher ADHD prescribing rate among boys compared to girls [7]. This gender distribution trend is evident among international literature. It is recognised that ADHD is often underdiagnosed and undertreated in females due to gender-based diagnostic biases and the tendency for girls to exhibit less disruptive, more inattentive symptoms that are overlooked. Additionally, societal expectations and internalised coping strategies in females can mask symptoms, delaying recognition and treatment [17].

Methylphenidate was the most frequently prescribed stimulant in our cohort with Concerta XL (n = 348, 20.5%) being the most frequently prescribed formulation of methylphenidate reported. This is consistent with previous research conducted by both Boland et al. and Mac Avin et al. which indicated methylphenidate to be the most frequently prescribed ADHD medication in Ireland [6, 18]. The study also described a significant increase in methylphenidate prescription over the study period between 2005 to 2015 [18]. In our cohort, atomoxetine (n = 116, 6.8%) was the most frequently prescribed non-stimulant medication followed by guanfacine (n = 61, 3.6%) and clonidine (n = 9, 0.5%). The high frequency of methylphenidate prescription compared to other ADHD medications can be attributed to its status as first line medication for children aged 6 to 17 years of age diagnosed with ADHD [19]. Broadly, the prescribing indication, and doses were within national and international guideline recommendations. The median starting and maintenance doses of stimulants reported in this data adheres to standard prescribing guidelines in Ireland with the recommended starting dose of methylphenidate immediate release (IR) 5 mg once daily or twice daily or alternatively methylphenidate extended release 5 mg once daily. Doses can then be increased in 5 mg increments at no more than weekly intervals, with ongoing monitoring of physical parameters after each dose increments along with review assessment of symptoms and side effects. The median maintenance doses for methylphenidate stimulants across all formulations in this cohort do not exceed the daily maximum licensed doses for children as follows: Concerta XL 54 mg, Ritalin LA 60 mg, Equasym XL 60 mg and Medikinet XL 60 mg [20]. The median dose for lisdexamfetamine, a second-line medication licensed for the use in the paediatric population when clinically unresponsive with optimum doses of methylphenidate, was also noted to be within the maximum daily licensed dose of 70 mg [21]. The third-line medication as described in guidelines is the non-stimulant medication, atomoxetine, with maximum daily licensed dose of 100 mg for children aged 6 to 17 years of age [22]. Guanfacine, the second most prescribed non-stimulant in our study, is licensed in children aged 6 years and above indicated where stimulants are not suitable, not tolerated or ineffective with doses dependant on age and weight [23]. Our study described a minimal amount of the use of clonidine (n = 9, 4.6%), a non-stimulant that is not licensed for the use of children and is primarily indicated for severe hypertension [24]. International guidelines state that clonidine may be prescribed off-label for ADHD with comorbidities including sleep disturbances, rages or tics, although they would require advise from a tertiary ADHD service [19].

Considering our findings from an international perspective, we highlight lower levels of polypharmacy in comparison to other international jurisdictions e.g., United States [25], and in our study the main medication prescribed alongside an ADHD related medication was melatonin. This is in contrast to data in the United States that highlight that up to 20.5% of patients may be prescribed ADHD polypharmacy (i.e., two ADHD related medications combined) and up to 40.7% may be prescribed psychotropic polypharmacy [25]. Peak initiation of ADHD-related medication in our study was within the 11–13 year age range and more male skewed (approximately 3:1), this is in contrast to recent Norwegian registry data where male-to-female ratios narrowed to parity in mid-adolescence, possibly highlighting ongoing under-recognition of girls with ADHD in Ireland [26]. From a dose perspective, our findings demonstrate that the median dose of methylphenidate lies comfortably (20-40 mg per day) within the international range, in comparison to higher maintenance doses in the United States (e.g., > 36 mg) and Japan (e.g., < 27 mg) [27]. We acknowledge that while it is important to make international comparisons for learning and quality control, within Ireland there is limited licensed medication for ADHD (as described in this study), in comparison to other jurisdictions (e.g., United States with greater than 25 approved formulations [28]. It is possible, the lower licensed medication in Ireland may limit clinicians ability to prescribe off-label if clinically indicated. This is evident again by our finding of less than 0.5% guanfacine prescribed in our study, which may be due to guideline/licensing of guanfacine as a second-line medication and highlights how regulatory landscapes shape national prescribing profiles [28].

The findings of our study must be interpreted within the context of the study limitations. Firstly, This study was limited to prescriptions issued within CAMHS and did not capture prescribing by other healthcare professionals, such as paediatricians, neurologists, or general practitioners. Secondly, As data collection was performed locally by multidisciplinary teams, there is a possibility of input inaccuracies or inconsistencies across services. The diagnosis classification used was not requested by services, although it is the International Classification of Disease-11 (ICD-11) or Diagnostic and statistical manual of mental disorders-5 (DSM-5) that is used in the Irish context. Data on non-pharmacological interventions were not collected. Several participant demographic factors were not collected, and information on prior medication exposure or whether prescriptions represented new initiations or renewals was not available. The closed-question format used in the audit tool limited the depth of information gathered about prescribing context, including details of side-effect monitoring. We did not have a sufficient sample size in the off-label group to complete meaningful subgroup analyses (e.g., gender or age differences), despite this, future research with larger off-label sampling sizes may help to understand underlying associations with off-label use. With the available dataset, we were not able to determine the tolerability and clinical impact of ADHD prescribing practices in CAMHS. This is deserving of future research and including in any future national audit of CAMHS prescribing.

## Conclusion

To the best of our knowledge, this is the first study that describes ADHD medication prescribing practices in children and adolescents attending mental health services in Ireland. This study demonstrates that ADHD prescribing practices in Ireland largely adhere to recommended guidelines, with minimal off-label use.

## Supplementary Information

Below is the link to the electronic supplementary material.

Supplementary file1 (DOCX 56 KB)

## Data Availability

The data is available by applying to the data controller (HSE).
