# Supplementary material for: ADHD prescribing: national findings of children and adolescents attending mental health services in Ireland
Source: Int J Clin Pharm. 2025 Sep 1;48(2):424–34. doi: 10.1007/s11096-025-01979-z (PMC12992343; doi:10.1007/s11096-025-01979-z)
Supplement: Supplementary file 1 — Supplementary file1 (DOCX 56 KB) [file 11096_2025_1979_MOESM1_ESM.docx]

**Title:** ADHD prescribing: national findings of children and adolescents attending mental health services in Ireland.

**Etain Cantwell^1*^ and Ivana Nelan^1*^, Sharifah Zahirah Idid^2^, Suzanne McCarthy^3 $^ and David O Driscoll ^1,2 $^.**

**^*^ contribute equally as first author(s).**

**^$^ contribute equally as senior author (s).**

**^1^ School of Public Health, Western Gateway Building, University College Cork, Cork, Ireland.**

**^2^ Specialist Neurodevelopmental ADHD Pathway, Cork and Kerry Mental Health Services, Cork, Ireland**

**^3^ School of Pharmacy, University College Cork, Cork, Ireland.**

**Corresponding author:**

**David O Driscoll –** [**davidodriscoll@ucc.ie**](mailto:davidodriscoll@ucc.ie)

**Supplementary Material**

**Table S1:** Summary of demographics of female participants prescribed a psychotropic, stimulant, or non-stimulant medication from July to December 2021 in Ireland.

**Table S2:** Summary of demographics of male participants prescribed a psychotropic, stimulant, or non-stimulant medication from July to December 2021 in Ireland.

**Figure S1:** Flow chart of analysed participants

**Table S1:** Summary of demographics of female participants prescribed a psychotropic, stimulant, or non-stimulant medication from July to December 2021 in Ireland (n=1,374)

|  | **Other Psychotropic** | | **Stimulant** | | **Non-stimulant** | | **p-value*** |
| --- | --- | --- | --- | --- | --- | --- | --- |
|  | (n=979) | | (n=342) | | (n=53) | |  |
| **Age categorised (years)** | **n** | **(%)** | **n** | **(%)** | **n** | **(%)** |  |
| 5-7 | 0 | (0) | 5 | (1.5) | 1 | (1.9) | <0.001 |
| 8-10 | 14 | (1.4) | 42 | (12.3) | 7 | (13.2) |  |
| 11-13 | 106 | (10.8) | 107 | (31.3) | 14 | (26.4) |  |
| 14-15 | 317 | (32.4) | 88 | (25.7) | 14 | (26.4) |  |
| 16-17 | 542 | (55.4) | 100 | (29.2) | 17 | (32.1) |  |
| **Categorised duration in service (years)** |  |  |  |  |  |  | <0.001 |
| 0-2 | 832 | (85) | 184 | (53.8) | 17 | (32.1) |  |
| 3-5 | 115 | (11.7) | 89 | (26) | 22 | (41.5) |  |
| 6-8 | 19 | (1.9) | 44 | (12.9) | 10 | (18.9) |  |
| 9-12 | 7 | (0.7) | 22 | (6.4) | 4 | (7.5) |  |
| >13 | 4 | (0.4) | 2 | (0.6) | 0 | (0) |  |
| Uncodable | 2 | (0.2) | 1 | (0.3) | 0 | (0) |  |
| **Consultant present on the team** |  |  |  |  |  |  | 0.23 |
| Yes | 917 | (93.7) | 314 | (91.8) | 51 | (96.2) |  |
| **Referral type** |  |  |  |  |  |  | <0.001 |
| Routine | 461 | (47.1) | 272 | (79.5) | 43 | (81.1) |  |
| Urgent | 513 | (52.4) | 68 | (19.9) | 8 | (15.1) |  |
| Missing | 5 | (0.5) | 2 | (0.6) | 2 | (3.8) |  |
| **Moderate - severe diagnosis** |  |  |  |  |  |  |  |
| Anxiety - Yes | 568 | (58) | 49 | (14.3) | 12 | (22.6) |  |
| ADD/ADHD - Yes | 63 | (6.4) | 337 | (98.5) | 50 | (94.3) |  |
| Depressive Disorder - Yes | 391 | (39.9) | 16 | (4.7) | 2 | (3.8) |  |
| Eating Disorder - Yes | 140 | (14.3) | 4 | (1.2) | 1 | (1.9) |  |
| OCD - Yes | 83 | (8.5) | 5 | (1.5) | 2 | (3.8) |  |
| psychosis - Yes | 18 | (1.8) | 0 | (0) | 0 | (0) |  |
| BPAD - Yes | 13 | (1.3) | 0 | (0) | 0 | (0) |  |
| Tourette's/Tics - Yes | 14 | (1.4) | 2 | (0.6) | 3 | (5.7) |  |
| **Medication prescription** |  |  |  |  |  |  | <0.001 |
| Methylphenidate | - | - | 290^1^ | (84.8) | - | - |  |
| Lisdexamfetamine | - | - | 39^2^ | (11.4) | - | - |  |
| Atomoxetine | - | - | - | - | 32 | (60.4) |  |
| Guanfacine | - | - | - | - | 17 | (32.1) |  |
| Clonidine | - | - | - | - | 3 | (5.7) |  |
| **Baseline physical parameters prior to medication** |  |  |  |  |  |  |  |
| No | 90 | (9.2) | 34 | (9.9) | 2 | (3.8) |  |
| Yes | 543 | (55.5) | 306 | (89.5) | 51 | (96.2) |  |
| Not applicable | 346 | (35.3) | 2 | (0.6) | 0 | (0) | <0.001 |
| **Monitor physical parameters during medication** |  |  |  |  |  |  |  |
| No | 90 | (9.2) | 21 | (6.1) | 2 | (3.8) |  |
| Yes | 499 | (51) | 318 | (93) | 51 | (96.2) |  |
| Not applicable | 390 | (39.8) | 3 | (0.9) | 0 | (0) | 0.001 |
| **Correspondence sent to family doctor (e.g., GP)** |  |  |  |  |  |  |  |
| No | 126 | (12.9) | 24 | (7) | 1 | (1.9) |  |
| Yes | 853 | (87.1) | 318 | (93) | 52 | (98.1) | 0.091 |
| **Follow up arranged** |  |  |  |  |  |  |  |
| Yes | 963 | (98.4) | 332 | (97.1) | 51 | (96.2) |  |
| **Abbreviations:** ADD attention deficit disorder, ADHD attention deficit hyperactivity disorder, OCD obsessive compulsive disorder, BPAD bipolar affective disorder, GP general practitioner (i.e., family doctor).  **^1^**Uncodable methylphenidate n=5 (9.4%)  ^2^ Uncodable lisdexamfetamine n=2 (3.8%) | | | | | | | |

**Table S2:** Summary of demographics of male participants prescribed a psychotropic, stimulant, or non-stimulant medication from July to December 2021 in Ireland (n=1,785)

|  | **Other Psychotropic** | | **Stimulant** | | **Non-stimulant** | | **p-value*** |
| --- | --- | --- | --- | --- | --- | --- | --- |
|  | (n=501) | | (n=1,144) | | (n=140) | |  |
| **Age categorised (years)** | **n** | **%** | **n** | **%** | **n** | **%** | <0.001 |
| 5-7 | 8 | (1.6) | 25 | (2.2) | 1 | (0.7) |  |
| 8-10 | 28 | (5.6) | 224 | (19.6) | 36 | (25.7) |  |
| 11-13 | 93 | (18.6) | 382 | (33.4) | 48 | (34.3) |  |
| 14-15 | 123 | (24.6) | 288 | (25.2) | 36 | (25.7) |  |
| 16-17 | 249 | (49.7) | 225 | (19.7) | 19 | (13.6) |  |
| **Categorised duration in service (years)** |  |  |  |  |  |  | <0.001 |
| 0-2 | 376 | (75) | 470 | (41.1) | 45 | (32.1) |  |
| 3-5 | 89 | (17.8) | 365 | (31.9) | 49 | (35) |  |
| 6-8 | 21 | (4.2) | 202 | (17.7) | 37 | (26.4) |  |
| 9-12 | 12 | (2.4) | 93 | (8.1) | 7 | (5) |  |
| >13 | 3 | (0.6) | 12 | (1) | 2 | (1.4) |  |
| Uncodable | 0 | (0) | 2 | (0.2) | 0 | (0) |  |
| **Consultant present on the team** |  |  |  |  |  |  | 0.73 |
| Yes | 472 | (94.2) | 1072 | (93.7) | 130 | (92.9) |  |
| **Referral type** |  |  |  |  |  |  | <0.001 |
| Routine | 272 | (54.3) | 1046 | (91.4) | 130 | (92.9) |  |
| Urgent | 226 | (45.1) | 91 | (8) | 10 | (7.1) |  |
| **Moderate - severe diagnosis** |  |  |  |  |  |  |  |
| Anxiety - Yes | 259 | (51.7) | 47 | (4.1) | 12 | (8.6) |  |
| ADD/ADHD - Yes | 100 | (20) | 1135 | (99.2) | 138 | (98.6) |  |
| Depressive Disorder - Yes | 162 | (32.3) | 15 | (1.3) | 4 | (2.9) |  |
| Eating Disorder - Yes | 15 | (3) | 1 | (0.1) | 0 | (0) |  |
| OCD - Yes | 60 | (12) | 6 | (0.5) | 1 | (0.7) |  |
| psychosis - Yes | 25 | (5) | 1 | (0.1) | 0 | (0) |  |
| BPAD - Yes | 2 | (0.4) | 0 | (0) | 0 | (0) |  |
| Tourette's/Tics - Yes | 9 | (1.8) | 7 | (0.6) | 4 | (2.9) |  |
| **Medication prescription** |  |  |  |  |  |  |  |
| Methylphenidate | - | - | 1000^1^ | (87.4) | - | - |  |
| Lisdexamfetamine | - | - | 111^2^ | (9.7) | - | - |  |
| Atomoxetine | - | - | - | - | 85 | (60.7) |  |
| Guanfacine | - | - | - | - | 44 | (31.4) |  |
| Clonidine | - | - | - | - | 6 | (4.3) |  |
| **Baseline physical parameters prior to medication** |  |  |  |  |  |  | <0.001 |
| No | 53 | (10.6) | 67 | (5.9) | 5 | (3.6) |  |
| Yes | 284 | (56.7) | 1059 | (92.6) | 133 | (95) |  |
| Not Applicable | 164 | (32.7) | 18 | (1.6) | 2 | (1.4) |  |
| **Monitor physical parameters during medication** |  |  |  |  |  |  | <0.001 |
| No | 48 | (9.6) | 45 | (3.9) | 3 | (2.1) |  |
| Yes | 280 | (55.9) | 1,097 | (95.9) | 136 | (97.1) |  |
| Not Applicable | 173 | (34.5) | 2 | (0.2) | 1 | (0.7) |  |
| **Correspondence sent to family doctor (e.g., GP)** |  |  |  |  |  |  | 0.008 |
| No | 57 | (11.4) | 78 | (6.8) | 12 | (8.6) |  |
| Yes | 444 | (88.6) | 1066 | (93.2) | 128 | (91.4) |  |
| **Follow up arranged** |  |  |  |  |  |  | 0.60 |
| Yes | 493 | (98.4) | 1120 | (97.9) | 137 | (97.9) |  |
| **Abbreviations:** ADD attention deficit disorder, ADHD attention deficit hyperactivity disorder, OCD obsessive compulsive disorder, BPAD bipolar affective disorder, GP general practitioner (i.e., family doctor).  ^1^ Uncodable methylphenidate n=24 (17.1%)  ^2^ Uncodable lisdexamfetamine n=7 (5%) | | | | | | | |

**Figures**

**X**

Met inclusion criteria

July – December 2021

**3 193**

Sample

**1 687**

Prescribed ADHD medication

**Anonymous details received:**

Gender, age, referral type, duration in service.

Known diagnoses.

Medication prescribed during July-December 2021 (name, target conditions, target symptoms if no target condition, starting dose and maintenance dose).

Prescribing standards.

**74**

Paediatric psychiatry services provided data

**J21 018**

Total population attending services

**Figure S1:** Flow chart of analysed participants
